# Gastric TFF1 Expression from Acute to Chronic *Helicobacter* Infection

**DOI:** 10.3389/fcimb.2017.00434

**Published:** 2017-10-09

**Authors:** Roberta Esposito, Silvana Morello, Megi Vllahu, Daniela Eletto, Amalia Porta, Alessandra Tosco

**Affiliations:** ^1^Department of Pharmacy, University of Salerno, Fisciano, Italy; ^2^PhD Program in Drug Discovery and Development, University of Salerno, Fisciano, Italy

**Keywords:** *Helicobacter* infection, trefoil factors, TFF1, cytokines, acute infection, chronic infection

## Abstract

TFF1, a mucin-associated secreted peptide of gastric mucous cells, is known as a protective agent for stomach epithelium under different stimuli, but its role upon *Helicobacter* infection is still not clear. In this paper we characterized TFFs expression, with particular attention to TFF1, under *Helicobacter* infection in gastric cell lines. A mouse model was used to distinguish TFF1 mRNA expression between acute and chronic stages of *Helicobacter* infection. Our results show that TFF1 expression is induced in infected cells; in addition, the inflammatory response upon *Helicobacter* infection is inversely associated to pre-existing TFF1 protein levels. In infected mice, TFF1 is initially upregulated in gastric antrum in the acute phase of infection, along with IL-1β and IL-6. Then, expression of TFF1 is gradually silenced when the infection becomes chronic and IFN-γ, CXCL5, and CXCL15 reach higher levels. Our data suggest that TFF1 might help cells to counteract bacteria colonization and the development of a chronic inflammation.

## Introduction

*Helicobacter pylori* is a spiral-shaped, Gram-negative bacterium which colonizes the human gastric mucosa of 50–75% of population all over the world (Calvet et al., 2013). *H. pylori* infection can cause chronic gastritis in most infected individuals and more severe pathologies in a subset of them, including neoplasia, B-cell lymphoma of mucosal-associated lymphoid tissue (MALT lymphoma) and invasive gastric adenocarcinoma (Cover and Blaser, 2009).

Acute infectious symptoms (nausea, halitosis, dyspepsia, and malaise) tend to resolve within 2 weeks. The poor evidence related to acute infection is from old cases of deliberate ingestion (Marshall et al., 1985; Morris and Nicholson, 1987) or some more recent groups of enrolled volunteers (Graham et al., 2004; Nurgalieva et al., 2005). Acute infection is accompanied by severe gastritis, characterized by infiltration of neutrophils and inflammatory cells. Within the first 14 days of infection a reduction in stomach acid secretion is described (Sobala et al., 1991), together with polymorphonuclear cells infiltration and interleukin 8 (IL-8) induction in gastric biopsies (Graham et al., 2004).

On the other hand, a persistent infection brings to a state termed “chronic superficial gastritis” (Warren, 2000), characterized by different types of tissue-infiltrated leukocytes (lymphocytes, macrophages, neutrophils, mast cells, and dendritic cells). Gastritis severity increases with the level of inflammation, but the pattern of inflammation determines the disease outcome. Host genetic factors, bacterial virulence, environmental factors, and age of infection all together influence the distribution of resulting gastritis (Robinson et al., 2007).

*H. pylori* infection has been associated with increased risk of developing gastric cancer (GC) together with genetic basis, environmental and nutritional factors. GC is the third most common cause of cancer-related death in the world (Ferro et al., 2015).

Gastric adenocarcinomas are classified according to prognosis into two main groups: early and advanced. Histologically, the most frequently used system is the Lauren classification, which recognizes two main subtypes: intestinal and diffuse (Lauren, 1965). The intestinal subtype is the most frequently diagnosed in older individuals, males more than females, strongly associated with *H. pylori* infection and it is characterized by malignant epithelial cells that show cohesiveness and glandular differentiation infiltrating the stroma; while the diffuse subtype is more aggressive, generally diagnosed in younger patients (Piazuelo and Correa, 2013).

Even if *H. pylori* association with intestinal metaplasia is, at this point, assessed, initial molecular gastric perturbation that could take into account subsequent malignant transformation have not been elucidated.

Recently, Trefoil Factor1 (TFF1) role has been studied during *H. pylori* infection. TFF1 is a mucin-associated secreted peptide that, together with the other two members of the Trefoil Factor Family (TFF2 and TFF3), participates in gastrointestinal mucosa repair (Kjellev, 2009). TFF1 is primarily secreted by the normal stomach mucosa and it is widely considered a gastric specific tumor suppressor (Xiao et al., 2015). Indeed, more than 50% of gastric cancers show a reduced TFF1 expression due to some point mutations (Park et al., 2000), promoter polymorphic variants (Moghanibashi et al., 2012), but mostly to loss of heterozygosity (LOH) and hypermethylation of its promoter (Carvalho et al., 2002).

Trefoil factor family proteins are differently expressed along the gastrointestinal tract; at the stomach level, TFF1 is mainly expressed in the fundus and antrum of gastric mucous cells; TFF2 in the mucous neck cells of fundic glands and basal cells of antral and pyloric glands, as well as duodenum (Brunner's gland) (Aihara et al., 2017); while TFF3 is undetectable in normal gastric mucosa, but strongly expressed in goblet cells of intestinal metaplasia (Im et al., 2013). Looking at the phenotype of genetically modified animals, TFF1 knock-out mice displayed hyperplastic gastric epithelium and 30% of them developed multifocal intraepithelial or intramucosal carcinomas (Lefebvre et al., 1996), while mice lacking TFF2 showed only a minimal phenotype with a slight reduction in proliferation rates in gastric mucosa (Farrell et al., 2002). Nonetheless, *H. pylori* infected TFF2-deficient mice developed more advanced premalignant lesions of atrophy, metaplasia and dysplasia than wild-type (Peterson et al., 2010). In addition, TFF3 knock-out mice showed impaired intestinal epithelial regeneration after injury (Mashimo et al., 1996). These observations, together with clinical data, led to consider TFF1 as a specific gastric tumor suppressor, although recent studies clarified that its expression is reduced only in the intestinal-type gastric cancers (Im et al., 2013), contrary to patients with diffuse and undifferentiated cancer type characterized by high TFF1 expression (Ren et al., 2006). The other two Trefoil factors are not considered tumor suppressors, even if lack of TFF2 seems to be predisposal to gastric malignancies and TFF3 is strongly up-regulated in intestinal metaplasia and therefore considered a marker of poor prognosis (Im et al., 2013).

The few data on TFF1 expression in biopsy specimens from *H. pylori* positive compared to negative patients are controversial: Kato and coworkers report no significant change in TFF1 expression (Kato et al., 2004); while other evidence show TFF1 reduction in *H. pylori* positive patients compared to the uninfected, both at the mRNA (Tomita et al., 2011) and protein levels (Van De Bovenkamp et al., 2005). On the other hand, cellular studies report an increase of TFF1 mRNA expression (Matsuda et al., 2008).

Recently, it has been shown that reconstitution of TFF1 expression in GC cells reduces *H. pylori*-induced inflammation (Soutto et al., 2015a) and *H. pylori*-induced activation of oncogenic β–catenin (Soutto et al., 2015b), suggesting a protective role of the trefoil peptide in *H. pylori*-induced chronic inflammation and carcinogenesis. Nonetheless, data on TFF1 role in prodromal phases of infection prior its chronic conversion are missing. More robust evidence are needed for the other two Trefoil Factors (TFF2 and TFF3) as well, also because a better picture of their expression levels could represent a specific hallmark for gastrointestinal pathologies and likely have prognostic value.

In this paper we characterized TFFs expression, with particular attention to TFF1, under *Helicobacter* infection in gastric cell models and in mice antrum. Our results demonstrate that TFF1 expression level in infected cells is inversely associated to the degree of inflammation. Interestingly, in infected mice, TFF1 is upregulated in the early phase of infection, while it is gradually silenced right after the acute injury phase.

## Materials and methods

### Cell cultures

The cell lines used in these experiments were: AGS (gastric adenocarcinoma, intestinal type, highly differentiated), KATO III (gastric carcinoma, derived from metastatic site, poorly differentiated), AGS-AC1, a TFF1 inducible hyperexpressing clone described previously (Tosco et al., 2010).

AGS were cultured in Ham's F-12 (Nutrient Mixture F-12 Ham, Euroclone), KATO III in RPMI 1640 (Euroclone), AGS-AC1 clone in DMEM (Euroclone), all media were supplemented with 10% (v/v) fetal bovine serum (FBS, Euroclone), 100 U/ml penicillin and 100 μg/ml streptomycin (Euroclone). Medium for AGS-AC1 was supplemented with 600 μg/ml G-418 (Sigma) and TFF1 expression was induced with 1 μg/ml of doxycycline.

All cell lines were maintained at 37°C in a 5% CO_2_ atmosphere.

### Bacterial cultures

*H. pylori* P12 strain, obtained from a German patient with a duodenal ulcer (Schmitt and Haas, 1994) kindly provided by Dr. Marguerite Clyne (University College Dublin) and *Helicobacter felis* strain ATCC 49179, were cultured on selective Columbia agar (Oxoid) containing 7% (v/v) defibrinated horse blood (Oxoid) and an antibiotic mix (DENT or Skirrow supplement respectively, Oxoid). Bacteria plates were incubated for 3–4 days in a capnophilic atmosphere (10% CO_2_) as suggested by (Park et al., 2011). For infection experiments, bacteria were scraped from confluent plates using brain heart infusion broth (BHI Difco) supplemented with 7% FBS (v/v). Cultures were assessed for motility and the bacterial optical density at 600 nm (OD_600_) was measured considering: 1 OD_600_ = 1 × 10^8^ bacteria/ml.

### *Helicobacter pylori* colonization experiments

For each cell line, optimal colonization conditions were determined using different MOIs (multiplicity of infection) and times of infection. Briefly, AGS and KATO III were seeded at 80% of confluence, after 24 h cells were washed three times with PBS to remove medium containing penicillin and streptomycin, and then infected with different concentrations of bacteria and for different times at 10% CO_2_. AGS-AC1 clone was seeded at 60% of confluence, after 24 h TFF1 expression was induced with doxycycline and, after 48 h of induction, cells were infected in medium without antibiotics and incubated at 10% CO_2_. For AGS and KATO III MOI 1:30 and 36 h of incubation were selected as optimal infection conditions, while for AGS-AC1 clone MOI 1:150 and 36 h of infection were selected for all further experiments.

Cultures were washed different times with PBS to remove non-adherent bacteria and then harvested with Trypsin 0.05%-EDTA 0.02% and used for RNA, DNA or protein extraction. For each condition, experiments were performed at least in triplicate.

### Real time PCR analysis

Total RNA was extracted using TRI Reagent (Sigma), 2 μg of total RNA was reverse transcribed into cDNA with M-MLV Reverse Transcriptase (Gene Spin S.r.l) and Real-Time PCR was performed using the Light Cycler 480 II instrument (Roche). Suitable dilutions of cDNA were used for each gene in a 12 μl reaction using Light Cycler 480 Probes Master and Real Time Ready Catalog Assay primers (Roche) for human TFF1 (Conf. number 100027378), human TFF3 (Conf. number 100064753) and human HPRT1 (Conf. number 100027387) following the manufacturer instruction protocol. While for all the other human (IL-8; IL-6; TFF2) and mouse (TFF1; TFF2; TFF3; IL-6; IFN-γ; IL-1β; CXCL-15; CXCL-5; HPRT1) genes, Light Cycler 480 SYBR Green I Master was used, human primers are listed in Supplementary Table 1 and mouse primers in Supplementary Table 2. Results were analyzed using the Delta-Delta CT method and HPRT1 as reference gene.

### Western blot analysis

The supernatants were clarified by centrifugation at 10,000 g for 10 min at 4°C. To obtain intracellular proteins, cell pellets were incubated in ice-cold lysis buffer (0.1% NP-40 in PBS 1X) for 30 min, sonicated and then centrifuged at 10,000 g for 10 min to remove cellular debris. Five micrograms of intracellular proteins and 20 μl of medium containing extracellular proteins were diluted with Laemmli buffer and subjected to western blot analysis using a polyclonal anti-TFF1 antibody (Tosco et al., 2010) and an anti-tubulin or anti-β actin antibody (Santa Cruz). Chemiluminescent signals of positive bands were detected by ImageQuant LAS 4000 (GE Healthcare, Waukesha, WI, USA) digital imaging system and quantified by ImageQuant TL software.

### Ethics statement

All animal experiments were approved by the Italian Ministero della Salute (Aut. n° 714/2015-PR) and performed according to institutional animal care guidelines (Italian Law 26/2014 based on the European Community Law for Animal Care 2010/63/UE).

### Mice infection

All studies were performed on female C57BL/6 mice (6 weeks old) obtained from Charles River Laboratories International, and maintained in a pathogen-free room. Mice were maintained in groups of five in microisolator cages with *ad libitum* access to food and water and were fasted overnight prior to infection and samples collection. Animals were infected by oral gavage with *H. felis* (10^7^ total bacteria per mouse in 100 μl) three times every other day. Control mice received 100 μl of medium. Mice were then maintained on fasting for 5 h after each inoculation. Control group (10 mice) and infected groups were sacrificed by cervical dislocation at 1, 3, 5, 10, 14, and 42 days post-infection (6 mice for day 14 and 8 mice for all the other groups). The stomach was removed and washed with PBS. For molecular analyses antrum was divided in three parts: one was immediately stored in RNA isolation reagent (TRI Reagent, Sigma), frozen in dry ice and stored at −80°C for Real Time experiments; the other two pieces were frozen in dry ice and used for DNA and protein analyses. For histologic analyses, stomachs were cut along the greater curvature, washed with PBS and laid flat on a cutting board. After fixing in a solution of 0.5% PFA, 0.5% Triton and 0.02% NaN_3_ for 2 h, tissues were incubated in 15% sucrose overnight at 4°C.

### Histologic analysis

After sucrose treatment, tissues were longitudinally dissected into three strips so that each fragment contained gastric cardia, body and antrum. The strips were embedded in OCT (Optimal Cutting Temperature) compound in cryomolds, placed on side to insure representation of all layers from mucosa through serosa in the final stained specimen, sectioned (5-μm slices) and stained with Hematoxylin and Eosin (H&E). The histopathological features of tissues were assessed by light microscopy (Axioplan 2 Imaging Universal Microscope with an AxioCam camera, Zeiss). Histologic scoring criteria of mouse gastritis were assigned according to Rogers (2012).

### Immunofluorescence analysis

Immunofluorescence analyses were performed on stomach tissue sections for the detection of F4/80 (rat anti-F4/80 PE conjugate 1:100; EBioscience cod. 12-4801-82), GR-1 (rat anti-GR-1 1:100, EBioscience cod. 14-5931-85) and TFF1 (rabbit anti-TFF1 1:500, LSBio cod. LS-C155659) proteins. Sections were fixed for 20 min in 4% paraformaldehyde, permeabilized with 0.5% Triton in PBS for 5 min then blocked with 20% goat serum in PBS + 0.5% Triton for 30 min followed by labeling with primary antibody overnight at 4°C. For GR-1 and TFF1 detection, sections were then washed and incubated, respectively, with an Alexa Fluor 488 goat anti-rat and an Alexa Fluor 594 goat anti-rabbit secondary antibody (1:5000) for 2 h at RT protected from the light. Visualization of the nuclei was achieved by Hoechst staining (1:5000, Invitrogen). Sections were mounted with 40% glycerol in PBS. Images were collected using an LSM 510 Laser-scanning Confocal Microscope (Zeiss).

### Statistical analysis

The results are expressed as means ± SEM or ± *SD* as appropriate. All statistical differences between two groups of data were evaluated by Student's *t*-test. Statistical analyses and graphing were done using PRISM4 software (GraphPad Software, La Jolla, CA, USA). A *p* < 0.05 was considered statistically significant.

## Results

### *H. pylori* infection induces TFF1 expression in gastric cancer cells

To better understand the role of TFF1 during *H. pylori* infection, we analyzed two cancer cell lines with significant difference in TFF1 basal levels: AGS, a gastric adenocarcinoma cell line, highly differentiated, intestinal type, which shows very low expression of TFF1, almost undetectable at protein level; KATO III, a signet ring cell carcinoma from a metastatic site, poorly differentiated, with higher expression of the protein (Supplementary Figure 1).

Firstly, we optimized the infection conditions by using different MOIs and different time points for bacterial incubation. Based on IL-8 induction, a cytokine up-regulated upon *H. pylori* infection (Lee et al., 2013), and a microscopic observation of cellular health, we selected the MOI 1:30 and 36 h of incubation as IL-8 was significantly increased and cellular shape was preserved; conversely, at higher MOIs and prolonged incubation time, both cellular lines showed suffering signals (Supplementary Figure 2).

**Figure 1:**
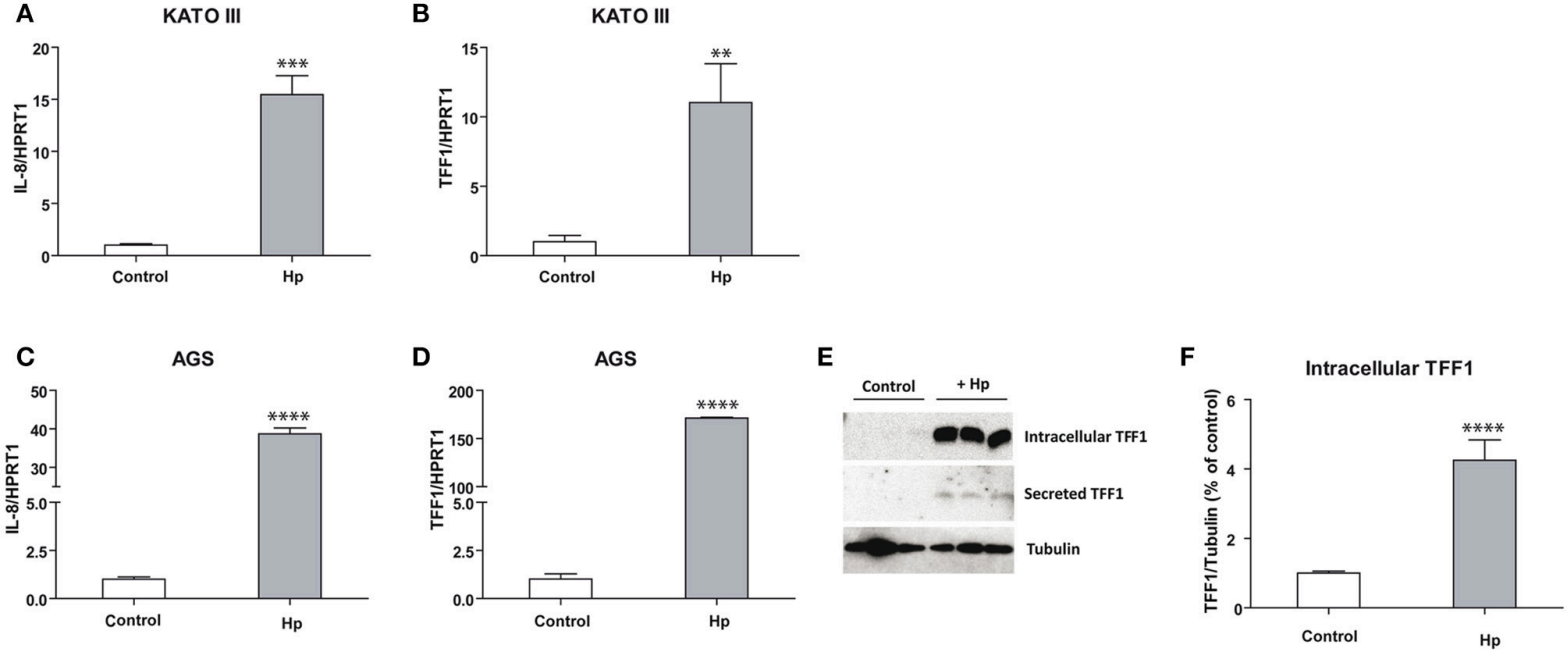
TFF1 and IL-8 transcriptional regulation upon *H. pylori* infection in KATO III and AGS cells. Real Time PCR analysis of IL-8 **(A–C)** and TFF1 **(B–D)** in KATO III and AGS gastric cancer cells after 36 h of *H. pylori* infection. **(E)** Western blot analysis of intracellular and secreted TFF1 protein in AGS cell line. **(F)** Densitometric analysis of intracellular protein signals. Experiments were carried out at least in triplicate and data are expressed as mean ± *SD* (*t*-test, ^**^p ≤ 0.01, ^***^p ≤ 0.001, ^****^p ≤ 0.0001).

As shown in Figures 1A–C, IL-8 is more induced in AGS (ca. 40-fold) than in KATO III (ca. 15-fold) cells after *H. pylori* infection, suggesting that the basal higher level of TFF1 in KATO III might protect cells from the inflammatory process. On the other hand, TFF1 mRNA induction caused by bacterial infection is higher in AGS (ca. 150-fold) (Figure 1D) compared to KATO III cells (ca. 10-fold) (Figure 1B). As a consequence, in infected AGS cells TFF1 protein becomes detectable in either intracellular lysates or in supernatant media as secreted form (Figures 1E,F). These data suggest that cells respond to bacterial insult by producing TFF1 protein, likely to counteract the infection.

**Figure 2:**
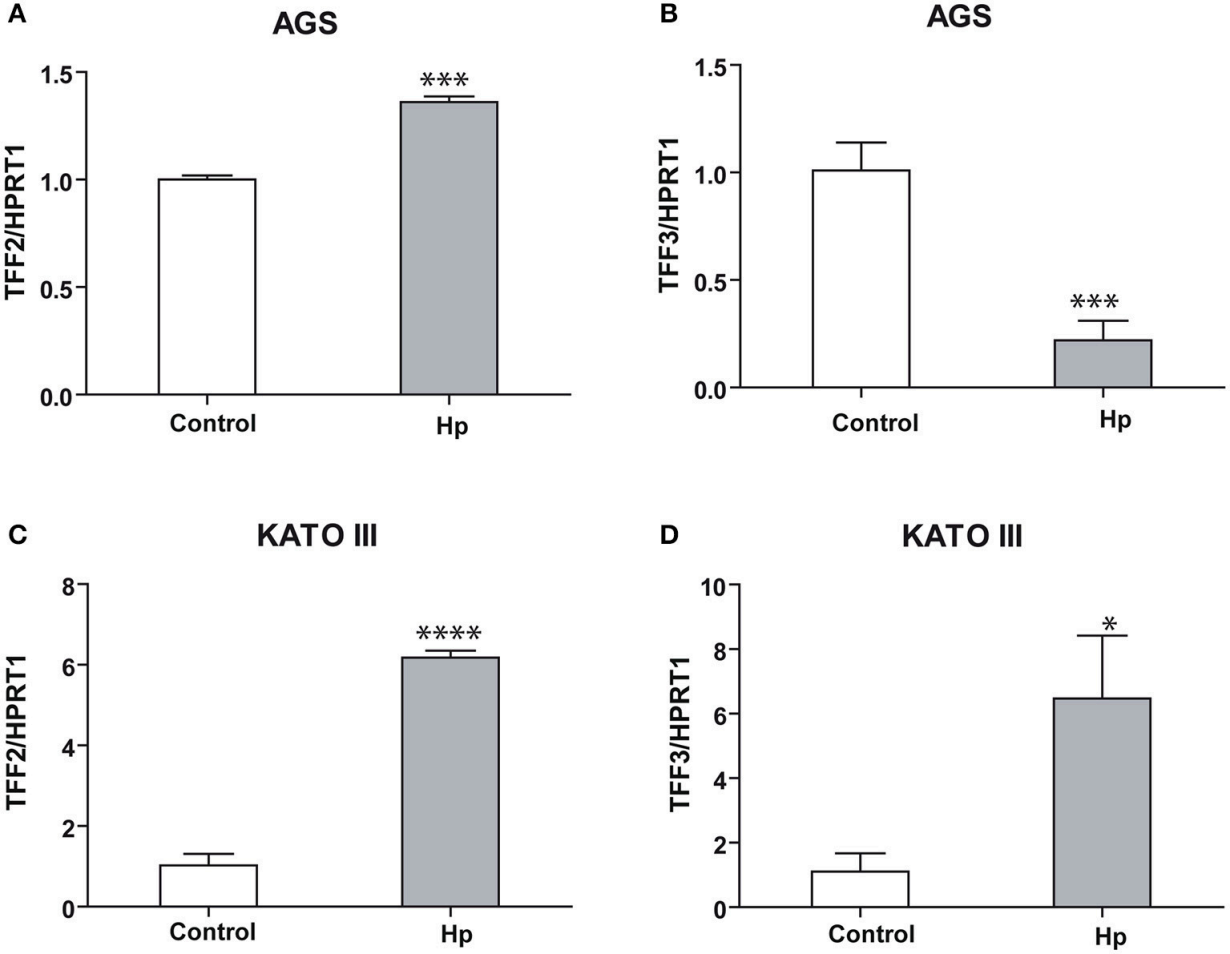
TFF2 and TFF3 transcriptional regulation upon *H. pylori* infection in KATO III and AGS cells. Real-time PCR analysis of TFF2 and TFF3 mRNA after 36 h of *H. pylori* infection in AGS **(A,B)** and KATO III cells **(C,D)**. Experiments were carried out at least in triplicate and data are expressed as mean ± *SD*. (*t*-test, ^*^p ≤ 0.05; ^***^p ≤ 0.001, ^****^p ≤ 0.0001).

Moreover, the analysis of expression level of the other two members of the Trefoil Factor Family, TFF2 and TFF3, produced different results in the two cellular lines. TFF2 and TFF3 are both induced in infected KATO III cells (Figures 2C,D), while in infected AGS cells TFF2 is slightly induced and TFF3 is strongly reduced (Figures 2A,B).

Taken together, these data suggest that TFF1 is induced upon *Helicobacter* infection in both cellular systems, while TFF2 and TFF3 responses change depending on the host system.

### TFF1 protein influences TFFs expression after *H. pylori* infection

Based on the previous results, we asked whether the different response to *H. pylori* infection was due to the different basal level of TFF1 in AGS and KATO III cells. To this aim, we decided to use an inducible hyper-expressing clone of TFF1, AGS-AC1, already characterized in our lab (Tosco et al., 2010), which expresses detectable level of TFF1 only under doxycycline control (Supplementary Figure 3).

As described previously, we optimized the conditions of infection, using different MOIs (1:60, 1:150, 1:300) for 36 h of incubation and selected MOI 1:150 which produced optimal IL-8 up-regulation without cell suffering (Supplementary Figure 4).

**Figure 3:**
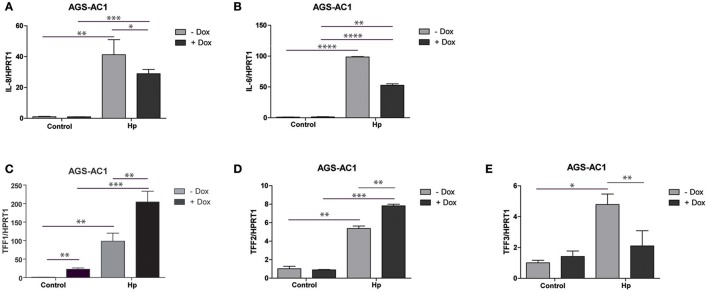
Trefoil factors and cytokines transcriptional regulation upon *H. pylori* infection in AGS-AC1 clone. Real-time PCR analysis of IL-8 **(A)**, IL-6 **(B)**, TFF1 **(C)**, TFF2 **(D)** and TFF3 **(E)** mRNA after 36 h of *H. pylori* infection in AGS-AC1 cells induced or not with Doxycycline to express TFF1. Experiments were carried out at least in triplicate and data are expressed as mean ± *SD* (*t*-test, ^*^p ≤ 0.05; ^**^p ≤ 0.01; ^***^p ≤ 0.001, ^****^p ≤ 0.0001).

As expected, bacterial infection caused IL-8 and IL-6 induction in both AGS-AC1 doxycycline- induced and -uninduced cells (Figures 3A,B). However, we observed a lower induction of both cytokines in hyper-expressing TFF1 AGS-AC1 cells compared to uninduced cells (Figures 3A,B).

TFF1 and TFF2 are up-regulated upon infection (Figures 3C,D), in both doxycycline-induced and -uninduced cells, but their levels are significantly higher in TFF1 hyper-expressing cells (dox-induced). On the other hand, TFF3 is up-regulated upon infection only in TFF1 not expressing cells (Figure 3E). The latter data are in contrast with what we observed previously in the aforementioned two different tumor cell lines (Figures 2B,D), likely because other regulatory mechanisms of TFF3 expression might occur in this system.

These results suggest that TFF1 protein is able to influence its own expression as well as TFF2 and TFF3 transcription, in response to outer insults like bacterial infection.

### TFF1 expression is differently regulated from acute to chronic *Helicobacter* infection

In order to verify whether this phenomenon is reproduced *in vivo* as well, we enrolled a mouse model (C57BL/6 strain) of *H. felis* infection. This system allows investigating TFF1 expression in either acute or chronic inflammation. Importantly, *H. felis* represents a better system compared to the adapted *H. pylori* SS1 strain, because it develops gastric adenocarcinoma through intestinal metaplasia and dysplasia mimicking humans infected with *H. pylori* (Hayakawa et al., 2013).

**Figure 4:**
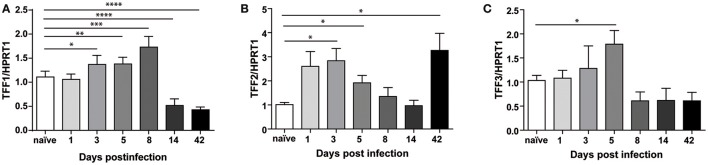
Trefoil Factors transcriptional regulation upon *Helicobacter felis* infection in C57BL/6 mice. Real Time PCR analysis of TFF1 **(A)**, TFF2 **(B)**, and TFF3 **(C)** mRNA in gastric antrum of C57BL/6 mice infected with *H. felis* and sacrificed at the indicated times (*d* = days; w = weeks) post-infection compared to naïve mice (n: 6–10 for each group). Data are mean ± SEM. (*t*-test, ^*^p ≤ 0.05, ^**^p ≤ 0.01; ^***^p ≤ 0.001, ^****^p ≤ 0.0001).

Mice were infected as described in methods section and sacrificed at different times, at 24 h after the first injection of the bacterial pathogen up to 6 weeks later (Supplementary Figure 5). The success of infection was assessed by PCR analysis of ureB gene (data not shown). Figure 4A shows that, in gastric antrum of C57BL/6 mice infected with *H. felis*, TFF1 mRNA is up-regulated at 3, 5, and 8 days post-infection (up to 2-fold compared to naïve), while it is already down-regulated after 14 days (0.3-fold compared to naïve) and remains low up to 6 weeks post-infection (42 days). At this longer time point we were also able to observe reduced TFF1 protein levels (Supplementary Figure 6).

Regarding the other two members of the family, we measured an up-regulation (up to 2 to 3-fold) at earlier time of infection and different behaviors after 6 weeks when TFF2 keeps increasing (Figure 4B), while TFF3 levels is lower (Figure 4C).

**Figure 5:**
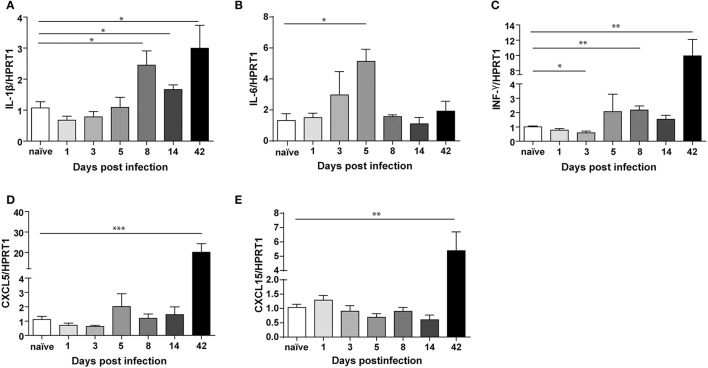
Transcriptional regulation of different cytokines upon *Helicobacter felis* infection in C57BL/6 mice. Real Time PCR analysis of IL-1β **(A)**, IL-6 **(B)**, INF-γ **(C)**, CXCL5 **(D)**, and CXCL15 **(E)** mRNA in gastric antrum of C57BL/6 mice infected with *H. felis* and sacrificed at the indicated times post-infection compared to naïve mice (n: 6–10 for each group). Data are mean ± SEM. (*t*-test, ^*^p ≤ 0.05; ^**^p ≤ 0.01; ^***^p ≤ 0.001).

In order to characterize the inflammatory response across the infection, we analyzed the levels of IL-1β, IL-6, IFN-γ, CXCL5, and CXCL15 (Figure 5), reported to be up-regulated upon *Helicobacter* infection in different studies (Schmitz et al., 2007; Figueiredo et al., 2014).

We measured significant levels (2 to 3-fold) of IL-1β (Figure 5A), at 8 days up to 6 weeks post-infection, and increased IL-6 levels (ca. 5-fold) at day 5 post-infection (Figure 5B). These cytokines are among the first ones produced during acute infection in humans (Graham et al., 2004). Moreover, we found a significant up-regulation of IFN-γ already at day 8 post-infection, which reaches higher levels at 6 weeks post-infection (Figure 5B).

On the other hand, CXCL5 (ca. 20-fold) (Figure 5D) and CXCL15 (ca. 5-fold) (Figure 5E) are strongly up-regulated only after 6 weeks, which marks definitely the transition from acute to chronic infection.

Our results demonstrate that TFF1 is up-regulated in the acute phase of infection together with IL-1β and IL-6, while is down-regulated in the chronic phase of infection (after 42 days) when IFN-γ, CXCL5, and CXCL15 increase.

**Figure 6:**
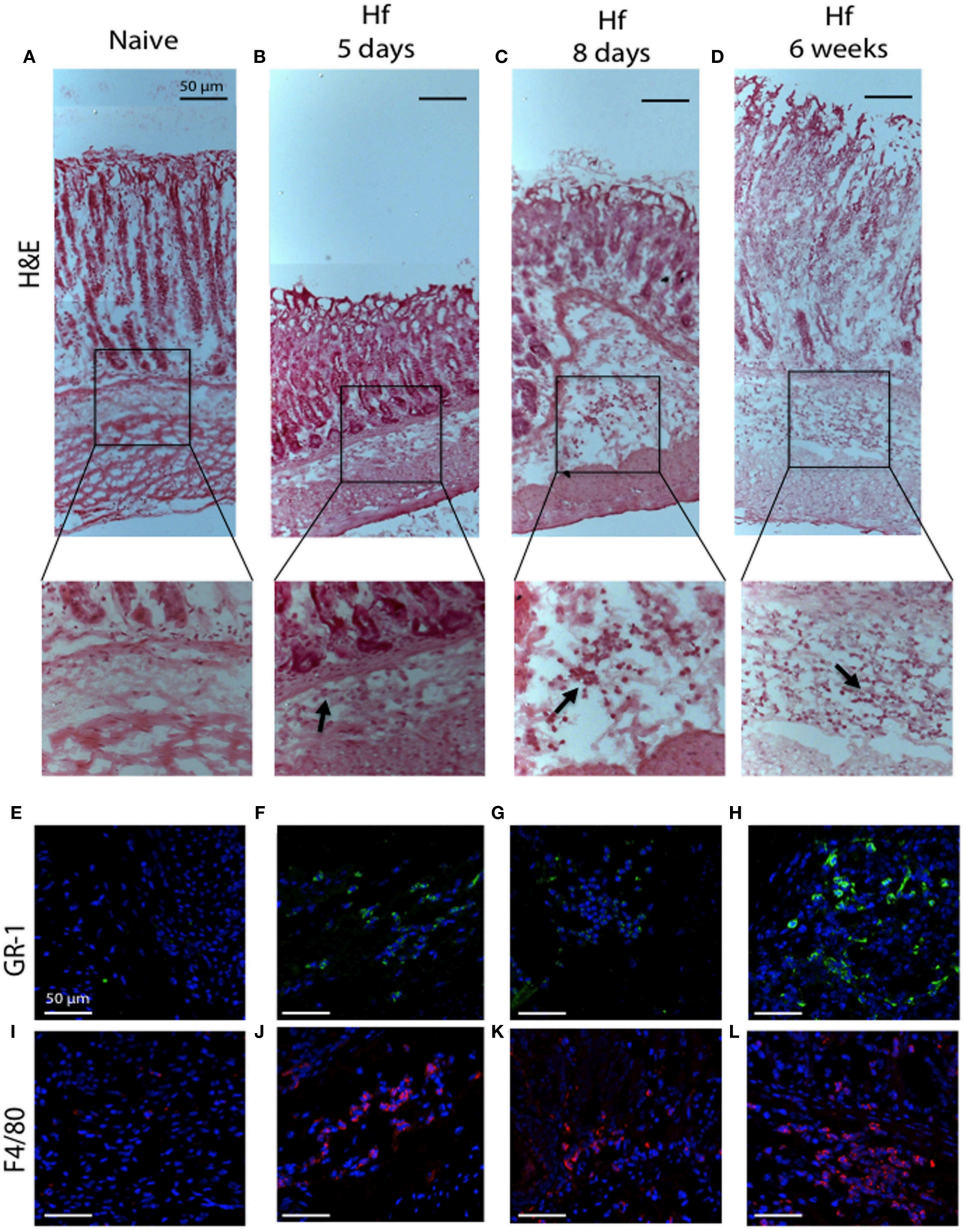
H&E and immunofluorescence analysis of C57BL/6 gastric corpus mucosae representative regions. **(A,E,I)** naïve mice; **(B,F,J)** 5 days; **(C,G,K)** 8 days; **(D,H,L)** 6 weeks *H. felis*-infected mice. Staining shows no significant pathological changes in naïve mice. Note the inflammatory cell infiltration in the submucosa (20x magnifications of boxed sections, black arrows) and the positive staining for GR-1 (green) and F4/80 (red) after 5 days and progressively more significant at 6 weeks post-infection. **(A–D)** 10x; **(E–L)** 40x. Hoechst for nuclei (blue). Scale bar: 50 μm.

Histologic observation of the entire stomach confirmed what previously measured. Hematoxylin and eosin (H&E) staining revealed essentially no morphologic changes in mice tissues at 5 and 8 days post-infection, yet clear signs of chronic gastritis across the entire tissues after 6 weeks of infection (Figure 6). In particular, mice at 6 weeks post-infection showed extensive infiltration of inflammatory cells at the level of mucosa and submucosa, across the entire tissue from the forestomach to pylorus, whilst samples from mice at 5 and 8 days post-infection showed less cellular infiltration with a specific localization in the *corpus* submucosa. Figures 6A,D reports representative images of cellular infiltrates in the *corpus*. Immunofluorescence analyses revealed the presence of F4/80-positive macrophages and Gr-1-positive neutrophils among the cellular infiltrates already at 5 days post-infection.

All together, the histological evidence accomplished the “inflammation criterion” and, according to the scoring criteria included in *Helicobacter* associated mouse gastric histology activity index (HAI) (Rogers, 2012), we assigned a score of 1 to samples at 5 and 8 days post-infection and a score of 2–3 to samples after 6-weeks since mice were infected.

These results corroborate previous observations and confirm that when the infection is acute (up to 8 days) and characterized by few infiltrates, cells respond increasing TFF1 levels. Conversely, during the chronic phase of infection (at 42 days) when cellular infiltrates increase, TFF1 expression is reduced.

## Discussion

TFF1 analyses on gastric biopsies from *Helicobacter* infected patients seem to indicate a reduced expression of the peptide, although data are limited (Van De Bovenkamp et al., 2005; Tomita et al., 2011) and restricted only to chronic infections. Regarding the other two members of the Trefoil Factor Family, gastric TFF2 is described to be silenced by promoter methylation during *Helicobacter* infection (Peterson et al., 2010), while no evidence is reported for TFF3 on gastric biopsies of *Helicobacter* infected patients. TFFs expression is always cross-correlated, the level of each of them changes depending on the specific gastrointestinal pathology and they might represent prognostic tools. Therefore, a more complete picture of TFFs expression is needed.

Here, to investigate TFFs expression, with particular attention to TFF1, under *Helicobacter* infection, we started using different cancer cells with different basal levels of TFF1. These systems were also useful to correlate the inflammatory response to the pre-existing TFF1 level. To better characterize the acute and chronic phases of infection and overcome technical limit due to inadequacy of cancer cell lines (Mustapha et al., 2014), we moved to a mouse model.

We chose three different cellular lines, KATO III, AGS and the inducible clone AGS-AC1: the first one is from a metastatic site, poorly differentiated, with high expression of TFF1 protein; the second one is from a gastric intestinal type, highly differentiated adenocarcinoma, with very low expression of TFF1; the last one is an hyper-expressing inducible clone of AGS. In all these three systems we observed a transcriptional induction of the peptide TFF1 after *H. pylori* infection, to the extent that TFF1 protein became detectable by Western blot analysis in AGS cells. The choice of the inducible clone was useful to discriminate the cellular response in presence or absence of TFF1 protein in the same cell line. Results from this cellular system showed that when the trefoil peptide is expressed, the pro-inflammatory cytokines (IL-8 and IL-6) induction upon infection is reduced. This result is in agreement with other data (Soutto et al., 2015a) and suggests that basal TFF1 level in the stomach could limit the inflammatory response, while a lower expression could lead to a more severe response.

Moreover, the presence of TFF1 might influence the regulation of TFF2 and TFF3 expression upon infection, since TFF2 turns to be more induced than expected, while TFF3 is not. The regulatory cross-talk among the three Trefoil peptides was already described (Taupin et al., 1999), nevertheless our analysis highlights such crosstalk upon *Helicobacter* infection. Interestingly, in hyper-expressing clone TFF1 is so strongly induced that its level cannot be explained as the mere sum of dox-induced plus *H. pylori-*induced mRNA; such result could be justified with an auto-induction mechanism, already described for TFF2 and TFF3 (Bulitta et al., 2002; Sun et al., 2016), and we are currently examining this possibility.

Taken together, our results on cellular systems suggest that during *Helicobacter* infection the pre-existing TFF1 favors the induction of TFF2 and TFF1 itself, known as gastric protective factors, while limits the up-regulation of TFF3, a negative marker in this district, as well as the cytokine response (IL-8 and IL-6).

However, our cellular data on TFF1 up-regulation upon *Helicobacter* infection, seem in contrast to *in vivo* analyses where it is down regulated (Van De Bovenkamp et al., 2005; Tomita et al., 2011). Hence, we based our *in vivo* experiments on the hypothesis that gastric mucosa, in the acute phase of infection, tries to face bacteria by up-regulating TFF1; when infection persists and becomes chronic, other molecular mechanisms occur and lead to the silencing of TFF1, along with other genes. To our knowledge, only Tomita and coworkers (Tomita et al., 2011) measured TFF1 upon *Helicobacter* infection in mice, but the analysis was carried out at 18 weeks post-infection, when relevant gastric damages have already happened.

For our *in vivo* experiments we used C57BL/6 mice infected with *Helicobacter felis*, since these model showed gastric metaplasia, dysplasia and invasive cancer mimicking *H. pylori* human infection, thus representing a better system than the *H. pylori* mouse adapted strain (Hayakawa et al., 2013).

We analyzed TFF1 mRNA in the very early phase of infection at 1, 3, 5, and 8 days post-infection and later at 2 and 6 weeks. We report that, during the acute phase of infection, TFF1 mRNA is significantly up-regulated in the *antrum* while, starting from 2 weeks post-infection, its level is lowered and at 6 weeks we observe an appreciable reduction of its protein signal. The other two members of the Trefoil Factor Family are both induced up to 5 days post-infection; then, their expression is back to basal levels and only TFF2 is again up-regulated at 6 weeks.

The analysis of mRNA level of different cytokines revealed a different pattern of induction: IL-1β, IL-6, and IFN-γ are up-regulated in the *antrum* of mice at early time of infection, while CXCL5 and CXCL15 are induced only at 42 days. Infiltrating macrophages and neutrophils start colonization of *corpus* submucosa at 5 and 8 days post-infection, extending to the entire stomach submucosa at 6 weeks.

Based on these data, we conclude that, in our experimental conditions, when TFF1 is up-regulated (acute phase of infection), there is an innate immune response which ends at 8 days post-infection, while when TFF1 is reduced (at 6 weeks) signs of the chronic phase of infection are visible. Therefore, we postulate that in humans with a compromised TFF1 expression, the inflammatory response might be more severe.

Recently, we have demonstrated that TFF1 promotes *H. pylori* colonization in cellular models probably through its binding with the rough form of bacterial lipopolysaccharide and that this effect is enhanced by copper (Montefusco et al., 2013). Copper was reported to promote the functional homodimeric form of TFF1 and its binding to form the active cuprocomplex (Tosco et al., 2010; Esposito et al., 2015). In the light of the present work, we postulate that TFF1 presence in the mucous layer, in spite of holding bacteria in the stomach, could block them in a less virulent form, protecting the underlying epithelium from a more severe inflammatory response.

In conclusion, in this study we demonstrated that TFF1 is up-regulated during acute *Helicobacter* infection and inversely correlated to inflammatory response, suggesting that it could help cells to counteract bacteria and the development of a chronic inflammation.

## Author contributions

SM, AP, and AT conceived and designed the experiments; DE, RE, SM, and MV performed the experiments, RE, SM, AP, and AT analyzed the data; AP and AT wrote the manuscript. All authors reviewed and approved the manuscript.

### Conflict of interest statement

The authors declare that the research was conducted in the absence of any commercial or financial relationships that could be construed as a potential conflict of interest.
