# Supplementary material for: Gastric TFF1 Expression from Acute to Chronic Helicobacter Infection
Source: Front Cell Infect Microbiol. 2017 Oct 9;7:434. doi: 10.3389/fcimb.2017.00434 (PMC5649190; doi:10.3389/fcimb.2017.00434)
Supplement: Supplementary file 1 [file DataSheet1.docx]

***Supplementary Material***

**Gastric TFF1 expression from acute to chronic *Helicobacter* infection**

Roberta Esposito^1†^, Silvana Morello^1†^, Megi Vllahu^1,2^, Daniela Eletto^1^, Amalia Porta^1*^, Alessandra Tosco^1*^.

^1^*Department of Pharmacy, University of Salerno, Fisciano (SA), Italy*

*^2^PhD Program in Drug Discovery and Development, University of Salerno, Fisciano (SA), Italy*

^#^These authors contributed equally to the research

^*^corresponding author:

Alessandra Tosco, Department of Pharmacy - Division of Biomedicine “Arturo Leone”, University of Salerno, Via Giovanni Paolo II, 132, Fisciano (SA), Italy

E-mail : [tosco@unisa.it](mailto:marzullo@unisa.it)

Tel: +39 089 969797

Fax: +39 089 969817

Amalia Porta, Department of Pharmacy - Division of Biomedicine “Arturo Leone”, University of Salerno, Via Giovanni Paolo II, 132, Fisciano (SA), Italy

E-mail : [aporta@unisa.it](mailto:marzullo@unisa.it)

Tel: +39 089 969455


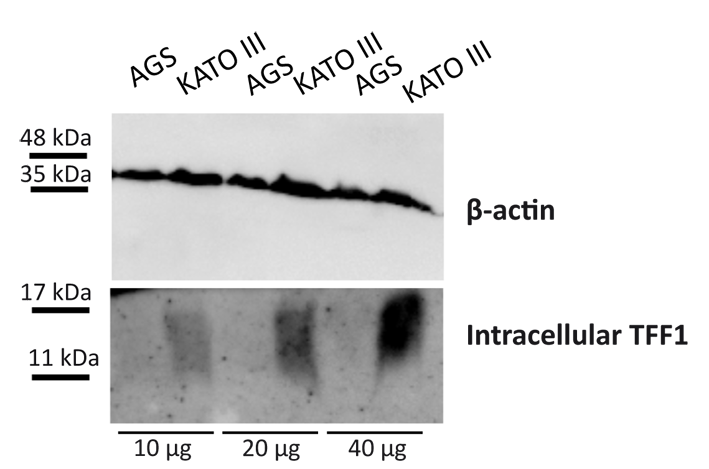


**Supplementary Figure S1.** Western blot analyses of intracellular TFF1 in AGS and KATO III cell lines. β-actin was used as loading control.

**Supplementary Figure S2**. Real-Time PCR analysis of IL-8 mRNA from KATO III and AGS control cells or after 36 and 72 h co-culture with *H. pylori* P12 strain. Results are expressed relative to controls using HPRT1 as reference gene.


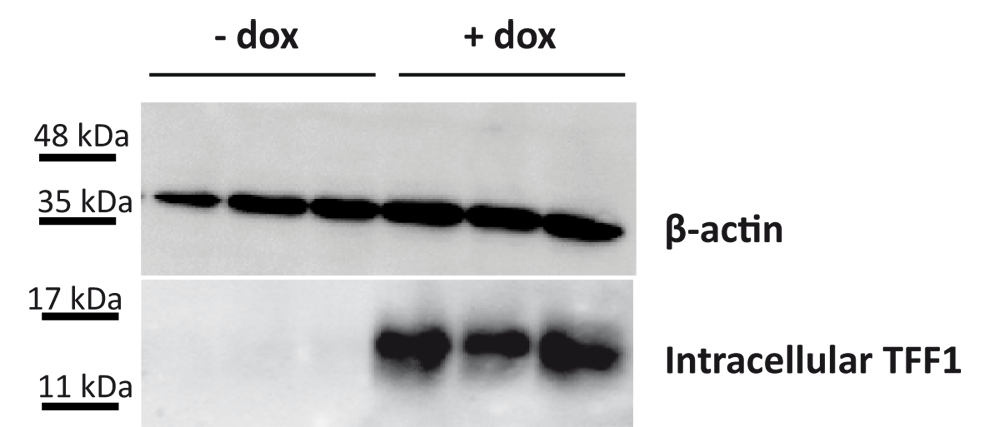


**Supplementary Figure S3.** Western blot analysis of intracellular TFF1 in AGS-AC1 cell line with or without Doxycyclin (Dox) to induce TFF1 expression. β-actin was used as loading control.

**Supplementary Figure S4.** Real- time PCR analysis of IL-8 mRNA in response to different *H. pylori* multiplicities of infection (MOI) in AGS-AC1 cell line. AGS-AC1 cell line with or without Doxycycline to hyperexpress TFF1 were incubated for 36 h with *H. pylori* P12 strain (MOI: 1:60; 1:150; 1:300).

**Supplementary Figure S5.** Schematic representation of the experimental design. The “Hf” bars indicate the time point of *Helicobacter felis* inoculation, while the “X” bars indicate the days of sacrifices .


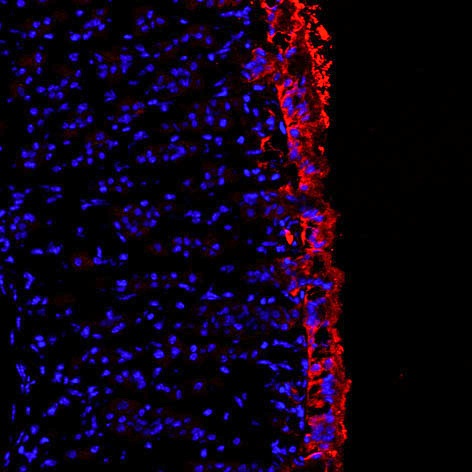

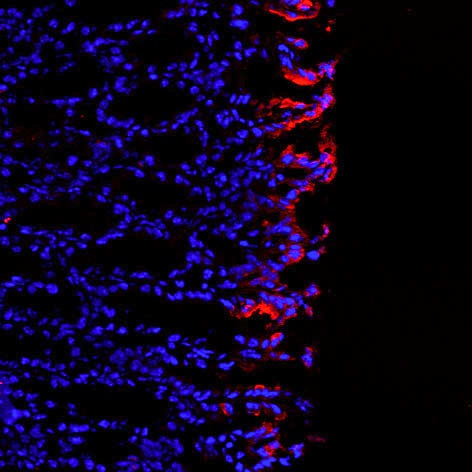


Naive

Hf

6 weeks

A

B

50 μm

**Supplementary Figure S6** Immunofluorescence analysis of C57BL/6 gastric mucosae. Representative regions of (A) naïve mice and (B) 6 weeks *H. felis*-infected mice. Red staining of TFF1 shows a reduction of protein levels in surface gastric epithelium of infected mice. (A-B; 20X. Hoechst for nuclei (blue). Scale bar: 50 μm).

| **Human primers** | **Sequences** | **T amp (°C)** |
| --- | --- | --- |
| **IL-8** | Fw 5’-ATGACTTCCAAGCTGGCCGTGGCT-3’  Rev 5’-TCTCAGCCCTCTTAAAACTTCTC-3’ | 60°C |
| **IL-6** | Fw 5’-AGTGAGGAACAAGCCAGAGC-3’  Rev 5’-GTCAGGGGTGGTTATTGCAT-3’ | 60°C |
| **HPRT1** | Fw 5’-GACCAGTCAACAGGGACAT-3’  Rev 5’-CCTGACCAAGGAAAGCAAAG-3’ | 60°C |
| **TFF2** | Fw 5’-CCAGTGCCTCCAGGCTGAG-3’  Rev 5’- AACCAGCCTCTCTTAGTAATGG-3’ | 60°C |

**Supplementary Table S1**. Human primers used for Real Time PCR analyses

| **Mouse primers** | **Sequences** | **T (annealing)** |
| --- | --- | --- |
| **TFF1** | Fw 5’- GGGATTCCCGTGGTGCTT-3’  Rev 5’-TGGACCTTAGAAGGGACATTCTTC-3’ | 60°C |
| **TFF2** | Fw 5’-GTCGAAACTGCTGCTTTTCC-3’  Rev 5’-TCGGCAGTAGCAACTCTCAG-3’ | 60°C |
| **TFF3** | Fw 5’-AGAGCCCTCTGGCTAATGCT-3’  Rev 5’-GCACCATACATTGGCTTGG-3’ | 60°C |
| **IFN-γ** | Fw 5’-TTCTTCAGCAACAGCAAGGC-3’  Rev 5’-ACTCCTTTTCCGCTTCCTGA-3’ | 63°C |
| **Il-1β** | Fw 5’-TTGTTGATGTGCTGCTGTGA-3’  Rev 5’-TGTGAAATGCCACCTTTTGA-3’ | 60°C |
| **IL-6** | Fw 5'-AGCCAGGTCCTTCAGAGAGAT-3‘  Rev 5'-GAGAGCATTGGAAATTGGGGT-3' | 60°C |
| **CXCL15** | Fw 5’-TTTGTTTGGATCCTGATGCTC-3’  Rev 5’- CGGTGGAA0ATTCCTTTTGTT-3’ | 60°C |
| **CXCL5** | Fw 5’-GCCCTACGGTGGAAGTCATA-3’  Rev 5’-TGCATTCCGCTTAGCTTTCT-3’ | 60°C |
| **HPRT1** | Fw 5’-TCAGTCAACGGGGGACATAAA-3’  Rev 5’-GGGGCTGTACTGCTTAACCAG-3’ | 60°C |

**Supplementary Table S2**. Mouse primers used for Real Time PCR analysis
